# Supplementary material for: Are carbon emissions trading and green financial instruments synergistic? -Comprehensive quantitative research based on content analysis
Source: PLoS One. 2024 Mar 7;19(3):e0298601. doi: 10.1371/journal.pone.0298601 (PMC10919601; doi:10.1371/journal.pone.0298601)
Supplement: S1 Data — (ZIP) [file pone.0298601.s001.zip › Data/地级市Z_F工作报告.zip!/╕ú╜¿/╚¬╓▌╩╨/└┤╫╘╣1⁄2╓┌║┼í╛┬φ┐╦╩2╛▌═°í┐_2011.docx]

各位代表：

　　现在，我代表泉州市人民政府向大会作工作报告，请各位代表连同《泉州市国民经济和社会发展第十二个五年规划纲要（草案）》一并审议，并请政协委员和其他列席人员提出意见。

　　一、“十一五”时期和2010年经济社会发展回顾

　　“十一五”期间，在市委领导下，各级政府各部门认真贯彻党的十七大精神，以邓小平理论和“三个代表”重要思想为指导，深入实践科学发展观，认真落实中央支持海西战略，有效应对国际金融危机冲击，扎实推进全面建设小康社会，较好完成“十一五”规划确定的目标任务。过去五年，是泉州经济大发展、大跃升的五年。2010年全市生产总值、财政总收入、一般预算收入、全社会固定资产投资分别达3564.76亿元、400.28亿元、181.53亿元和1250.81亿元，是2005年的1.98倍、2.6倍、2.4倍和3.5倍；2010年规模以上工业比重、重工业比重分别达88.6%、41.6%，比2005年高18个和3.2个百分点。荣获“转型·2010中国经济十大领军城市”、全国科技进步先进城市（七连冠）、中国优秀创新型城市等称号。晋江、石狮、南安、惠安、安溪五县（市）连续多年入围全国县域经济基本竞争力百强。过去五年，是泉州城乡大建设、大融合的五年。城市发展从沿江时代步入环湾时代，《泉州市城市总体规划（修编）》获省政府正式批准实施，中心市区建成区面积从2005年70平方公里，拓展到环湾区域建成区150平方公里；城镇化率从49%提高到60%。累计投入“三农”资金266亿元，创建各级新农村建设试点示范村889个，支持各级扶贫开发重点村370个。完成公路、电网、水利、通信投资分别达370亿元、100亿元、97亿元、113亿元，城乡基础设施大为改善。荣获国家环保模范城市、国家园林城市等称号，通过国家卫生城市复查。过去五年，是泉州对外大开放、大收获的五年。泉三高速公路、福厦铁路、斗尾30万吨级码头等重大交通设施投入运营，泉州港、肖厝港成为首批两岸海上直航口岸，泉州晋江机场获准对外开放，服务海西的东出西进综合通道初步建成。“十一五”期间累计完成外贸出口、实际利用外资、实际利用台资分别达289.9亿美元、103.7亿美元和8.5亿美元，是“十五”期间的2.8倍、2.1倍和2.8倍。被列为全国18个改革开放典型地区之一，荣获中国外贸百强城市、中国最具投资潜力50强等称号。过去五年，是泉州民企大成长、大提升的五年。我们始终坚持解放思想，保持政企一心、攻坚克难的好传统，特别是面对国际金融危机的严重冲击，连续召开4场千名企业家大会，动员全市上下坚定信心、顽强拼搏，民营企业在危机中发展壮大，民营经济地位更加突出。亿元企业、上市企业、驰名商标数量分别达1096家、61家和54件，比2005年增加713家、41家和38件。荣获中国十大品牌城市、中国品牌之都、国家商标战略实施示范城市等称号。过去五年，是泉州民生大投入、大改善的五年。2010年城镇居民人均可支配收入、农民人均纯收入分别达25155元和9296元，比2005年增长77%和51.8%。累计投入教育、医疗卫生、文化体育、社会保障分别达214.02亿元、50亿元、26.55亿元和60.42亿元，建成海峡体育中心、中国闽台缘博物馆、南音艺苑、梨园古典剧院等重大社会事业设施；成功举办第六届全国农运会、首届海峡两岸茶博会；闽南文化生态保护实验区获批设立，泉州南音入选人类非物质文化遗产代表作名录。荣获全国综治最高奖“长安杯”、中国十佳和谐可持续发展城市、全国双拥模范城（六连冠）、全国人民防空先进城市等称号。

　　2010年是“十一五”最后一年，我们围绕“两个加快”，按照市委“项目带动、转变提升、和谐进步、奋力发展”工作思路，全力推进科学发展、跨越发展，经济社会保持良好发展态势。一年来，主要工作和成效是：

　　致力打好“五大战役”，发展基础更加厚实。深入开展“项目建设年”活动，组织实施“五大战役”项目1628个，完成年度投资803.1亿元。重点项目建设战役，245个在建重点项目完成年度投资439.8亿元，其中泉厦和福泉高速扩建、石狮鸿山热电厂、晋江燃气电厂、500千伏泉州东通道工程、15个110千伏及以上输变电站等项目建成投入使用，泉州湾跨海大桥、泉三高速安溪连接线、莆永、仙金、晋石、南石高速公路及湄洲湾南岸铁路支线等项目开工建设，南惠高速、肖厝作业区5#和6#泊位工程、泉州晋江机场改建等项目加快推进。新增长区域发展战役，泉州湾、湄洲湾南岸、安永德三大增长区域完成年度全社会固定资产投资958.3亿元。城市建设战役，完成年度投资88.6亿元。小城镇改革发展战役，完成年度投资171.3亿元。民生工程战役，组织实施580个项目，完成年度投资63.7亿元。这些为泉州新一轮经济社会跨越发展打下良好基础。

　　致力转变发展方式，产业升级步伐加快。出台落实14个重点产业调整振兴实施意见，组织实施技改项目253个，新增亿元企业171家，形成纺织服装、鞋业、石油化工3个超千亿元产业集群，高新技术产业、重工业产值分别增长25%和38.8%；联合石化全面投产达产，海峡轨道客车、中国国际信息技术产业园、玖龙纸业等项目开工建设，中化炼油等项目加快推进，石狮PTA、钧石能源太阳能电池生产线等项目竣工投产；央企对接会签约项目16个，总投资1175亿元。强化企业自主创新，完成财政科技投入4.72亿元，微波通信产业集群获国家创新基金2623万元支持；新增高新技术企业61家、省级创新型示范企业26家；新增省级企业（工程）技术中心19家，产业技术创新战略联盟5家；新增中国驰名商标15件，国家重点新产品9项。参与各类标准制修订企业64家。成立科技创投基金2家。国家大学科技园福建分园6家企业投产，永春生物医药公共服务平台建设进展顺利。发挥人才支撑作用，制订我市中长期人才发展规划纲要，出台实施引进高层次创业创新人才等政策，安排3500万元人才开发专项经费；入选省级高层次人才（团队）4个，新增博士后科研工作站3家、院士专家工作站5家；成功举办“6·16”泉州人才交流大会。大力发展现代服务业，实现社会消费品零售额1202.91亿元，增长17%；完成港口货物吞吐量8455.37万吨和集装箱136.95万标箱；东海总部经济区确定15家入驻企业；泉州软件园确定10家入驻企业；完成旅游总收入252.6亿元，增长13.4%；实现金融机构存款余额3313.71亿元、贷款余额2717.12亿元，分别增长21.9%和25.8%。

　　致力发展现代农业，基础地位继续巩固。全市实现农林牧渔业产值232.11亿元。认真落实支农惠农政策，全年支农支出增长25.6%，兑现补贴资金2.32亿元。完成粮食种植面积任务，创建市级新农村建设试点示范村20个，抓好扶贫开发工作重点村20个。加快农业产业化步伐，新增国家农业产业化重点龙头企业1家，农业标准化示范区4个，农业“三品”认证48个，国家地理标志产品2个，农民专业合作社412家。成功举办第三届海峡两岸农产品采购订货会。获批成立国家级台湾农业技术交流推广中心，海峡两岸（安溪）茶产业合作示范区被确认为全国农产品加工创业基地。大力改善农村生产生活条件，湄洲湾南岸供水（二期）、崇武中心渔港等项目基本完工，23.5万农村人口饮水安全得到解决；提级改造农村公路100公里；完成130个村电气化建设；有效防抗台风及强暴雨，完成421户受灾农房建设；家园清洁行动扎实开展，完成改厕5.2万户，新增沼气用户6950户；完成“造福工程”搬迁8000人和地质灾害危险点搬迁319户；植树造林14.63万亩，治理水土流失20.08万亩。

　　致力拓展城市空间，城市功能日益增强。加快片区建设更新，编制完成中心市区城市景观、公交线网等专项规划；东海、城东、北峰、江南、晋江滨江等5个片区实现投资43亿元；龙头山、云山等8个旧城改造项目实现投资6.1亿元。万达城市综合体开工建设。加快完善市政设施，福厦高铁泉州站建成投入使用；站前大道、江滨北路改造基本完工，东海隧道、后渚至城东通道、站前大桥等项目建设加快；完成道路沥青化改造18.5公里；完成中心市区电力线路缆化52公里；新增绿地191公顷，城市景观整治有序推进。加快提升城市管理水平，建立国家卫生城市常态化管理机制，启动“数字城管”建设；整治城市机动车“冒黑烟”现象，500部新型环保公交车、400部出租车投入运营；建成30个社区服务中心，市区物业管理覆盖率达69.3%；遏制“两违”取得成效，市容市貌有所改观。加快推进小城镇建设，出台落实小城镇综合改革建设试点意见，确定3个省级、12个市级试点镇，完成试点镇总体规划修编，安排市本级专项补助金4500万元。新增城乡建设用地增减挂钩指标1593亩，有偿转让1502亩、交易金额3.07亿元。

　　致力开展环境整治，生态建设成效显著。强化节能降耗工作，列入国家级、省级节能扶持项目分别达18个和50个，淘汰落后产能企业1129家。强化水资源环境保护，扎实做好国家环保模范城市复查迎检；组织实施近海水域污染整治项目76个，完成投资8.88亿元，水质达标率75%；组织实施重点流域整治项目112个，完成投资10.6亿元；启动崇武至秀涂海岸带资源环境保护工作，顺利完成年度目标；率先成立水资源管理委员会，实行水资源统一规划、管理、调配和监控。强化环保基础设施建设，全市新增（修建）污水管道187.09公里，晋江（二期）、安溪和惠安垃圾焚烧发电厂投入运行，日污水、垃圾处理能力分别达62.25万吨和5450吨，城市（含县城）生活污水集中处理率和垃圾无害化处理率分别达80.5%和99.5%。强化企业环保责任落实，开展落实重点排污单位环境保护主体责任自查自律行动年活动，对1359家污染企业实施集中停产整顿。

　　致力推动泉台合作，改革开放大步迈进。不断深化体制改革，泉州开发区、高新区获批为国家级园区，洛江开发区实现扩区；修订出台推进企业改制上市工作意见，组织实施“121工程”，新增上市企业16家；出台鼓励金融业发展意见，新增商业银行分行4家，筹建泉州农村商业银行、安溪村镇银行；组建市交通运输集团和4个省级重点出口商品基地商会。不断深化泉台合作，泉州台商投资区管理机构挂牌成立；全市新批台资项目31个，实际利用台资2.37亿美元；4家企业赴台投资；泉州晋江机场成为两岸直航新航点，泉金航线客运量突破10万人次，对台海上货运直航实现常态化，泉澎签订交流合作协议11项。不断提升开放型经济发展水平，实际利用外资24.91亿美元，新批投资超千万美元项目100个，引进世界500强企业2家；全市进出口总额突破100亿美元，启动跨境贸易人民币结算试点工作；成功举办泉州商品博览会、“品牌泉州香江行”、赴台经贸文化交流活动及首届世界泉州同乡恳亲大会、世界泉商大会暨世界泉州青年联谊会。

　　致力建设和谐泉州，社会保持安定稳定。进一步加强劳动就业工作，全市新增城镇就业15.37万人，新增农村劳动力转移就业6.27万人，培训各类劳动者25.6万人次，城镇登记失业率1.18%。加快完善社会保障体系，参加职工“五险”人数增加34.26万人次，参加新农合人数、城镇居民医保人数分别增至548.6万人和161.37万人，新增低保参保人数0.45万人；新型农村社会养老保险试点工作扎实推进，职工医保实行市级统筹，制发社保卡202.2万张。坚持教育优先，全市11个县（市、区）全部通过省“双高普九”评估验收；泉州八中、泉州四中完成校园置换并分别合并到培元中学、泉州七中；盘活医高专、儿童发展职业学院旧校区，扩大市实验小学、幼师附属幼儿园等优质教育资源；中小学校舍安全工程开工建设面积112万平方米；完成创建义务教育阶段标准化学校306所；校园及周边安全有效加强。大力发展文化事业，成功举办首届闽南文化节、第六届中国航海日活动、第二届海峡印刷技术展览会；实施闽南文化生态保护区“十百千基础工程”，设立非物质文化遗产传习所50个、展示点199个；泉州中山路入选中国十大历史文化名街，泉州数字图书馆和18个乡镇综合文化站建成投入使用。加快医药卫生改革发展，组织实施9项基本、5项重大公共卫生服务项目，所有公立基层医疗机构都配备使用基本药物；7个县（市、区）实施药品零差率销售改革试点；市一院城东院区、传染病院投入使用，福医大附属二院东海分院、市三院新院建设顺利推进；重点支持边远困难乡镇卫生院建设，所有乡镇卫生院基本达到国家建设标准和省设备配置标准。“五位一体”的医疗纠纷处置机制基本形成。全市人口出生率11.18％。，政策符合率93.79%，入选全国流动人口计划生育基本公共服务均等化试点市；第六次人口普查顺利开展。加大保障性安居工程建设力度，完成住房公积金归集23.2亿元、发放20.68亿元；建设各类保障性住房和改造危旧房22580套（户）；东海保障性住房项目开工建设。扎实推进新一轮“平安泉州”建设，深化社会治安综合治理，依法严厉打击违法犯罪活动，做好信访和矛盾纠纷排查调处工作；基本完成企事业单位安全生产主体责任三年行动目标，安全生产事故四项指数全面下降；食品药品安全监管得到加强；全面完成“五五”普法工作。政府其他工作齐头并进，离退休干部服务、老龄、残疾人、妇女儿童、民族宗教、外事侨务、广播电视、信息化建设、国防动员、民兵预备役、支前、人防、统计、审计、物价、口岸、方志、档案、气象、防震减灾等工作取得新成绩。

　　致力推进依法行政，政府建设得到加强。自觉接受市人大及其常委会监督，认真执行市十三届人大七次会议和十四届人大五次会议议案决议，依法保障人大代表执行职务。支持市政协履行职能，主动接受政协委员民主监督。办理人大代表建议368件、政协委员提案453件，办复率均达100%，满意率分别达100%和99.78%。推进新一轮政府机构改革，49个工作机构缩减至40个。大力实施简政放权，159项市级审批事权下放各县（市、区），41个市直单位行政执法事项授权泉州台商投资区，部分县（市、区）行政审批事权下放乡镇。规范行政自由裁量权和行政指导工作扎实推进。深入开展“作风建设年”活动，推行三个“一条龙”审批服务，完善执法信息和网上审批平台，启动全程式网上招投标及远程评标。认真落实党风廉政建设责任制，加强反腐倡廉工作，处理违法违纪公务人员174人，挽回经济损失3292.93万元。圆满完成市委、市政府确定的11件27项为民办实事项目。

　　各位代表，“十一五”期间，我市经济建设和社会各项事业取得辉煌成就，这是在省委、省政府的正确领导下，市委统揽全局、科学决策，市人大、市政协大力支持、有效监督，全市人民和衷共济、团结奋斗的结果。在此，我代表市人民政府向全市广大干部群众，各民主党派、工商联、无党派人士、各人民团体和离退休老同志，驻泉省部属单位、驻泉部队、武警官兵，企业界人士、来泉创业务工人员及广大港澳台同胞、海外侨胞、海内外朋友致以崇高的敬意！对人大代表、政协委员和全市人民给予政府的信任和支持表示衷心的感谢！

　　当前，我市经济社会发展中也存在不少困难与问题，主要体现在：加快转变发展方式任务繁重，高新技术产业、服务业特别是现代服务业总量偏小，工业投资占全社会固定资产投资比重偏低；人才、劳动力、资金、土地、水资源等要素制约依然突出；节能减排、环境保护工作任重道远。中心城市辐射带动力不强，公共交通、城市景观、宜居水平有待提升，统筹城乡区域发展困难不少。社会事业发展相对滞后，民生保障水平有待提高，与人民群众的新期待仍有差距。出生人口性别比失衡未得到根本解决。食品药品安全工作还需加强，安全生产仍有隐患。社会调解机制和治安防控体系仍需完善。个别公务人员不作为、乱作为乃至腐败行为仍有存在。2010年度18个指标有2个未达到预期要求。对此，我们要增强责任感和紧迫感，采取有力措施，认真加以解决。

　　二、“十二五”时期主要目标任务

　　“十二五”时期，泉州发展仍处于大有可为的重要战略机遇期。新的科技革命和产业革命方兴未艾，国家确定新一轮发展宏伟蓝图，中央支持海西建设力度不断加大，ECFA的签署生效推动两岸经贸合作加速深化，为我市进一步拓宽发展空间创造有利条件。同时，必须清醒地看到，我市面临的国际国内竞争日益激烈。我们必须抢抓机遇，有为运作，在转变中实现跨越，在跨越中促进转变，真正实现科学发展、跨越发展。

　　根据市委十届八次全会通过的“十二五”规划《建议》，市政府编制“十二五”规划《纲要》（草案）。我市“十二五”发展的指导思想是：以邓小平理论和“三个代表”重要思想为指导，牢牢把握海西战略机遇，以科学发展为主题，以加快转变发展方式、加快建设经济强市、推动跨越发展为主线，以保障和改善民生为根本，加快农业现代化、新型工业化和城镇化，加快社会建设，深化改革开放，深化泉台交流合作，做大做强产业、港口、城市，持续推进海峡西岸经济区现代化工贸港口城市建设，实现全面建设小康社会，奋力开启建设现代化泉州新征程。

　　“十二五”时期，我市经济社会发展的总体目标和重要任务是：力争比全省提前2年、比全国提前5年实现全面建设小康社会，为2020年率先在全省基本实现现代化奠定基础。一要突出调整升级，奋力推动经济更好更快发展。围绕转变经济发展方式，加快发展现代农业、先进制造业、现代服务业、战略性新兴产业及海洋经济，实施“百千万亿”倍增计划、企业改制上市“121工程”和科技创新计划，打造世界纺织鞋服、石材产业基地和我国重要的石化、装备制造业、体育产业和光电信息产业基地，建设海西重要物流节点城市、贸易展览中心、金融服务中心，争创国家服务外包基地城市。围绕构筑现代基础设施体系，大力完善以港口为取向的综合交通体系，加快建设“智能泉州”，提高能源资源保障和防灾减灾能力。到2015年，全市生产总值超过6500亿元，年均增长11%以上，力争比2010年翻一番；财政总收入超过900亿元，年均增长18%以上，力争突破1000亿元；规模以上工业产值年均增长17%；三次产业比例调整为2.2∶57.8∶40，轻重工业比例调整为55∶45。二要突出统筹协调，实施城乡一体化发展。围绕做大做强海西中心城市， 统筹2980平方公里泉州大都市圈发展，突出“980”环湾区域规划建设，不失时机推进行政区划调整，大力提升城市功能，创新城市管理机制，推动环湾区域城市一体化发展。围绕统筹城乡发展，加快改善农村生产生活条件，完善农村发展体制机制，扎实推进小城镇建设，提升县域经济综合实力。力争到2015年，环湾区域建成区面积250平方公里。三要突出先行先试，不断增强发展的动力和活力。围绕构建有利于科学发展、跨越发展的体制机制，深化经济体制、行政体制及相关配套改革，进一步提升民营经济发展水平。围绕增创开放型经济发展新优势，转变外贸发展方式，提高招商选资质量，深化与港澳侨交流合作；拓展泉台合作空间，大力推动泉州台商投资区等园区创新发展。力争“十二五”期间，累计实际利用外资（历史可比口径）140亿美元，外贸出口年均增长10%。四要突出生态建设，打造资源节约型和环境友好型社会。深入贯彻节约资源和保护环境基本国策，全面推行节能减排，加强生态市建设，加快发展绿色经济，促进经济社会发展与人口资源环境相协调，增强可持续发展能力。五要突出民生优先，推动社会更加繁荣进步。围绕建设创新型城市，切实增强自主创新能力，打造海西人才高地，加快推进教育强市建设。围绕建设文化强市，提升人民群众文明素质和创造活力，加快发展文化事业，争创全国文明城市。围绕创建和谐幸福家园，积极促进就业创业，切实提高城乡居民收入，完善社会保障体系，提高全民健康水平，加强和创新社会管理，确保人民群众安居乐业。

　　三、2011年政府主要工作安排

　　2011年是实施“十二五”规划的开局之年，我市经济社会发展主要预期目标为：地区生产总值增长12.5%，力争达到13.5%；财政总收入增长17%，其中一般预算收入增长17%；规模以上工业产值增长19%；工业增加值增长13.4%；第三产业增加值增长12.5%；全社会固定资产投资增长21%；外贸出口增长8%；实际利用外资（历史可比口径）增长8%；社会消费品零售额增长16%；居民消费价格总水平涨幅控制在4%左右；城镇居民人均可支配收入增长10%，力争达到12%；农民人均纯收入增长10%，力争达到12%；城镇登记失业率控制在2%以内；人口自然增长率控制在7％。以内；完成省下达的节能减排任务。各级政府各部门要认真贯彻落实省委孙春兰书记、黄小晶省长来泉调研“晋江经验”的重要讲话精神，按照市委“项目带动、转变提升、和谐进步、跨越发展”工作思路，开拓进取，真抓实干，确保开好局、起好步。重点要推进八个方面工作：

　　（一）持续打好“五大战役”，深化扩大内需战略，进一步增强经济社会发展后劲

　　致力提高投资的速度和效益。启动实施“万亿固定资产投资”倍增计划，安排“五大战役”项目1741个，总投资7921亿元，年度投资1002亿元。重点项目建设战役要突出抓大项目、好项目，组织实施重点项目418个、年度投资610亿元以上，其中要全力确保278个在建项目完成年度投资533亿元以上。新增长区域发展战役要全力确保完成年度全社会固定资产投资1115亿元，其中泉州湾、湄洲湾南岸、安永德区域分别完成年度投资860亿元、210亿元、45亿元。城市建设战役要突出抓好专项规划、市容整治、城市交通、缆线下地、绿地系统、旅游景点、供水供气、污水处理、城市公厕、城市综合体等10项工作，组织实施项目196个，确保完成年度投资139亿元；小城镇改革发展战役要在抓好6个省级和9个市级试点镇的基础上，新筛选确定一批市级试点镇，着手实施项目660个，确保完成年度投资195.6亿元。民生工程战役要大力实施项目467个，确保完成投资57.61亿元。认真落实“五个一”工作机制，全力做好用地、融资等要素保障工作，加强土地管理，提高供地率。致力提高消费的能力和层次。积极构建扩大消费需求的长效机制，不断提高居民收入水平，进一步释放居民消费潜能；推广安溪引进新华都在乡镇建设商业综合体的做法，扶持发展大型购物中心、集贸市场和社区便利店，新增便民商业网点96个，深化实施“万村千乡市场工程”；继续开展家电下乡及以旧换新工作；积极发展新兴消费业态，培育体育健身、旅游休闲、假日节庆等消费热点，促进消费结构升级；重视物价调控，稳定消费价格总水平。

　　（二）夯实农业农村基础，推进小城镇改革发展，进一步建设农民幸福生活的美好家园

　　健全强农惠农机制。认真落实中央1号文件精神，按照“大兴水利强基础、狠抓生产保供给、力促增收惠民生、着眼统筹添活力”的要求，做到“三个重点、三个确保”。市、县两级财政安排支农资金17.12亿元。完善农村土地承包关系，深化集体林权制度改革，发展农业农村政策性保险。加强农村“七大员”队伍建设管理。加大重视扶持老少边岛发展。大力发展现代农业。坚持“抓龙头、建基地、打品牌、拓市场、深加工、扶协会”，启动农业产业化“131强龙工程”，加强“五新”推广，扶持155家市级农业龙头企业，建好茶叶等8大类特色农产品加工集聚区，新增农产品“三品”认证22个，争创国家级、省级农业产业化示范区，创建一批“五有”农民专业合作示范社。完成粮食种植面积254万亩，抓好“菜篮子”工程，建好53个省级副食品调控基地、64个无公害生产基地和泉州肉品加工厂。抓好重大动植物疫病防控，健全农产品质量安全监管和可追溯体系。提升新农村建设水平。抓好20个市级新农村建设试点示范村和10个省级、20个市级扶贫开发重点村及5个旧村居改造试点村建设，继续实施“造福工程”和地质灾害危险点居民搬迁项目。大力发展农村社会事业，让农民也能享受一些城市的文化生活。加快水利改革发展，大力推进山美水库、桃源水库扩蓄和晋江、石狮引水工程，认真实施晋江流域岸线整治、中小河流治理及水库、水闸、海堤除险加固等水利项目，配合做好省北水南调工程前期工作，完善防汛抗旱减灾体系。治理水土流失面积15万亩。建好梅林、东埔一级渔港。完成农村公路提级改造100公里。实施新一轮农网升级改造工程。推广“世纪之村”信息服务平台。深入开展家园清洁行动，实施农村“厕所革命”和沼气工程。加快小城镇建设步伐。做好省、市级试点镇规划工作，用足各项优惠政策和专项资金，引入民间资本参与开发，谋划实施一批带动性好的生产性项目、基础设施项目、宜居综合体项目，打造一批各具特色的宜居宜业示范镇。

　　（三）围绕转变发展方式，强化人才创新引领，进一步增强现代产业核心竞争力

　　组织实施“百亿企业倍增计划”和“千亿产业集群倍增计划”，加快重点产业调整振兴步伐，确保工业固定资产投资增长25%以上，占全社会固定资产投资的比重达44%以上，创建国家级、省级新型工业化产业示范基地。力促传统产业高端化。围绕整合提升纺织鞋服、建筑建材、食品饮料、工艺制品等产业，组织实施技术改造项目200个以上；发挥“泉州市质量奖”导向作用，扩建茶叶“国家质检中心”，筹建水暖洁具、石材建陶“国家质检中心”，支持企业主导制修订各类标准；规划建设体育用品园区，鼓励泉州高校发展服装、鞋类等专业；合理引导企业兼并重组、强强联合，打造一批核心竞争力强的大企业、大集团。力促成长型产业集群化。加快泉港、泉惠石化园区建设，上游抓好中化炼油、联合石化“脱瓶颈”、福建炼化二期等项目，下游抓好石狮PTA二期、福橡合成橡胶、百宏化纤等项目，形成完整石化产业链。加快晋江、南安装备制造业基地建设，积极发展造船修船、汽车、轨道机车、工程机械、轻纺机械等成套装备，提升装备制造业配套能力。建好鲤城、南安、惠安等光电基地，做好国家创新基金微波通信产业集群试点工作，抓好钧石能源、金保利等项目，壮大光电信息产业集群。加快建设中国包装印刷产业（晋江）基地，培育绿色造纸印刷产业。力促高新技术产业化。大力发展节能环保、生物与新医药、新能源、新材料等战略性新兴产业，推广物联网、云计算等新一代信息技术运用。认真实施《进一步鼓励企业自主创新的若干意见》，组织开展“企业创新三年行动”，市、县两级财政安排科技支出4.49亿元；实施科技计划项目150项，抓好“6·18”签约项目跟踪落实，组建产业技术创新联盟5家，争创省级以上研发机构10个，确保新增高新技术企业30家；推动泉州高校与产值亿元以上企业开展战略协作；争创国家知识产权示范市，确保实现全国科技进步先进城市“八连冠”。认真落实我市中长期人才发展规划纲要，实施12项人才重点工程；完善人才政策，用好人才开发专项经费；在东海保障性住房中预留200套作为引进人才公租房；建好用好博士后科研工作站、院士专家工作站；设立引进高层次人才“一站式”服务窗口，加强非公企业人才职称评定工作，引导高校毕业生面向企业就业；稳定现有人才队伍。力促第三产业规模化。强化对总部经济、物流、金融等现代服务业发展的政策引导，抓好五大总部经济区项目，海峡箱包物流商城、江南商贸物流中心、洛江仓储配送中心、石湖港石材物流园区等物流项目，泉州软件园、中国国际信息技术产业园等信息化项目。大力发展文化旅游业，支持清源山争创国家5A级旅游景区，稳妥有序推进泉州古城、崇武古城保护性开发，建好南少林寺片区、安溪海峡茶博园、永春北溪旅游区、中国国际瓷都印象生态园等项目，培育发展锦绣庄等5个文化创意产业园。力促招商选资多元化。用好泉州开发区、高新区、出口加工区等平台，跟紧央企对接会签约项目，力争有7个项目落地建设；加强针对世界500强的招商，提升“9·8”等签约项目履约率；精心组织参加省里在香港、澳门的招商活动，以及在北京召开的大型民营企业制造业项目洽谈会；深入实施“回归工程”，规划建设“上市公司创业园”，有效引导我市境外上市企业返程投资。力促内外市场协调化。发挥我市品牌众多优势，引导企业抱团入驻国内主要城市大型专业市场，加快专卖店、专柜布点，推广电子商务和网络营销，提高国内市场占有率。积极转变外贸发展方式，用足跨境贸易人民币结算试点、出口信保、境外参展等外贸促进政策，建好4个省级重点商品出口基地，延伸加工贸易增值链，提高自主品牌和高附加值产品出口比重，加快发展服务贸易，有为应对国际贸易摩擦，全力拓展南美等新兴市场。深化与香港、澳门经贸联系；进一步发挥侨资侨力作用；做好国际友城工作，促进国际经济技术合作。力促改革攻坚纵深化。出台推进民营经济“二次创业”的政策措施。规范用好融资平台。支持银行业金融机构发展，发挥好为我市建设融资的主体作用；推动泉州银行、石狮农村合作银行跨区域发展，推动农村合作银行、信用联社组建农村商业银行，积极引进金融机构，发展村镇银行、小额贷款公司。新增境内外上市企业15家以上。总结推广企业建造员工公寓、一线员工股权激励等做法，有效破解用工难题。

　　（四）突出港口龙头作用，构建现代综合运输体系，进一步提升基础设施保障能力

　　港口方面要围绕打造主枢纽港，重点加快石湖5#和6#泊位、肖厝5#和6#泊位、中化配套码头、秀涂“人工岛”起步工程和16#泊位等项目进度；继续落实港口生产优惠政策，着力培育国际航线，加快港后区和晋江“陆地港”建设，发展第三方、第四方物流，力争港口货物、集装箱吞吐量分别达到9300万吨和150万标箱；争取设立泉州保税港区。高速公路方面要围绕构建“一环两纵三横五联”网络，重点确保南惠高速通车，泉州湾跨海大桥、莆永、金安、仙金、晋石、南石、安溪连接线等项目完成年度计划。铁路方面要围绕构建“三纵一横五联”网络，重点加快湄洲湾南岸支线、福厦铁路惠安站建设，做好长泉、秀涂港口支线、中化泉州石化专用线等项目前期工作。空港方面重点要加快泉州晋江机场改建。能源方面重点要抓好南埔电厂（二期）、惠安电厂和一批输变电工程等项目。同时，大力推进外走马埭垦区回填和围头湾、石井围垦启动工程，增强用地保障能力。

　　（五）更加注重宜居品位，加快同城化步伐，进一步做大做强环湾中心城市

　　开展“城市建设管理年”活动，研究落实环湾同城化具体举措。优化城市空间结构。认真实施《泉州市城市总体规划（修编）》，通盘考虑城市轴线、景观和建筑风貌，确保环湾各片区控制性详规、专项规划无缝对接。推进东海、城东、北峰丰州、江南、晋江滨江、石狮蚶江、台商投资区等片区全面开发，完成市行政中心搬迁工作。畅通环湾路网通道，确保东海隧道、站前大桥、刺桐路改造、坪山路新建高架桥等项目竣工，加快泉州火车站综合客运交通枢纽、后渚至城东通道、田安大桥等项目建设，推进南北迎宾大道、普贤路至中心市区、万虹公路等项目改造，做好城市轨道交通前期工作。有序开发城市地下空间，重点解决地下管网和停车场问题。创新土地节约集约利用机制，用好城乡建设用地增减挂钩政策，提高土地利用率。塑造城市优美景观。建好万达等城市综合体，抓好环湾39个“三旧”改造项目；高标准建好东海滨江和观音山、北峰丰州滞洪区等城市公园，筹划实施清源山至观音山休闲绿道工程；全面开展北渠综合整治，推进泉南水厂三期、金鸡水厂、城市规划建设成就展示馆、泉州新工人文化宫及东湖、西湖音乐喷泉等项目建设。提升城市精细化管理水平。切实整治交通拥堵现象，优先发展公共交通，构建环湾公交体系；强化城市管理综合执法，坚决制止“两违”；预防和治理“城市病”，有效解决尾气、油烟、噪音、工程车滴洒漏等问题；加快“数字城管”建设；探索社会管理创新工作，发挥社区作用，加强社区管理，创建和谐社会。

　　（六）充分发挥海西效应，注重经贸交流实效，进一步开创泉台合作新局面

　　加速泉州台商投资区开发开放。积极争取先行先试政策，高起点、前瞻性编制各项规划。全面铺开基础设施建设，确保南北主干道建成通车，抓好杏秀路拓改、产业园区“七通一平”等项目，力争完成固定资产投资70亿元以上。加快海峡轨道客车、玖龙纸业、天岗精密机械等重大产业项目建设。高起点做好城市服务区规划建设，有序推进洛阳江、百崎湖、泉州湾整治开发。建立紧密经贸合作关系。做好ECFA早收清单和后续补充协议有关内容对接。主动衔接台湾六大产业振兴方案和四项智能型产业计划，深度对接台湾电子、光电、机械、石化等产业，力争在引进现代物流、信息服务、金融等现代服务业上取得突破。做好台资企业转型升级试点城市工作。不断扩大泉金客运和泉澎、泉台货运直航规模，拓展对台小额贸易，办好第四届海峡两岸农产品采购订货会，努力把我市建设成为对台民生产品重要出口基地、台湾农产品物流基地和大陆台资企业原辅材料、零部件配送中心。支持企业上岛投资。不断拓宽对台交流领域。办好第二届闽南文化节，继续实施一批泉台高校合作办学项目，积极引进台湾管理、营销、研发等方面高层次人才，开展乡镇结对交流，发挥好中国闽台缘博物馆功能，深化两地民间文化旅游往来。

　　（七）坚持绿色低碳发展，落实节能环保责任，进一步提高生态文明水平

　　落实节能减碳。深化十大节能工程，组织实施5个国家级重大节能项目及20个省级、30个市级重点节能和循环经济项目，基本完成重点耗能企业能耗计量数据采集和联网工作，坚决淘汰落后产能。推广使用清洁能源和煤炭清洁高效利用技术，抓好小岞风电场、绿谷金太阳示范电厂等项目建设。认真落实绿色信贷政策，推广合同能源管理，探索碳交易试点和低碳产品认证。充分发挥水资源管理委员会职能作用，建设节水型城市。落实治污减排。深化工业园区环境整治，加大印染、制革、电镀、重金属等行业及污染集控区深度治理。完成南安石材集中区建设，推动晋江石材行业整体退出。加强环保设施建设运营，筹建餐厨垃圾处理中心，抓好污水管网、生活垃圾转运站、南安垃圾焚烧发电厂（二期）等项目及107个公厕建设。深入开展重点排污单位环境保护主体责任自查自律行动年活动，严格污染物排放标准和环境影响评价。落实生态治理。认真落实市十四届人大五次会议议案决议，加大崇武至秀涂海岸带资源环境保护力度；认真执行市十三届人大七次会议议案决议，持续推进近海水域环境污染治理；完善“两江”上游水资源保护补偿机制，沿江两岸乡镇都要规划建设污水处理设施；有效解决重点流域及城市内沟河、泉州湾南岸十一孔桥闸、安平桥上游及小流域等环境污染问题。推进农村生态环境连片整治。加快溢油应急设备库建设，提升海域船舶污染应急处理能力。组织实施泉州湾河口湿地自然保护区红树林生态修复工程。围绕建设“森林泉州”，实施“四绿”工程，植树造林30万亩，加强森林防火和“青山挂白”治理，为子孙后代留下清洁空间。做好迎接国家环保模范城市复查工作。

　　（八）着力保障改善民生，全面推进社会建设，进一步建立健全基本公共服务体系

　　提高教育现代化水平。认真开展义务教育学校标准化建设，抓好城东、东海学园和七中江南新校区等项目；保障来泉务工人员随迁子女平等接受义务教育权利；深入推进素质教育，启动“教育强县”创建工作。推进各类教育协调发展，实施学前教育三年行动计划，完成31所公办幼儿园建设任务；大力发展高中阶段教育，办好各类技工学校，组建职教集团2个；提升高校办学质量和发展水平，加强与华侨大学共建；鼓励规范民办教育发展；关心老年教育、特殊教育，构建学习型社会。加强校园安全工作，新开工建设中小学校舍50万平方米。提升群众健康水平。深化医药卫生体制改革，加快公立医院改革，抓好福医大附属二院东海分院、泉州医疗中心、中医联合医院及23个中心乡镇卫生院、13个社区卫生服务中心建设，加大医疗人才引进力度，全市基层医疗单位建立健全稳定长效的多渠道补偿机制，全面推广基本药物制度和药品零差率销售；做好重大传染病防控工作。贯彻落实《全民健身条例》，承办世界女排大奖赛，办好市第九届运动会。致力提高人口素质，稳定适度低生育水平，开展出生人口性别比失衡等专项治理，加强流动人口计生服务管理创新，充分发挥计生协会作用。实施新一轮妇女儿童发展纲要，关注留守儿童的健康和安全。大力繁荣文化事业。深入实施闽南文化生态保护区“十百千基础工程”，推动“海上丝绸之路”和“闽南红砖建筑”申遗；加快泉州木偶剧院、歌剧院及乡镇综合文化站、农家书屋、职工书屋等项目建设；鼓励文艺创作，加强文化市场监管；加快文化、广电资源融合发展。做好档案和第二轮修志工作。深化文明城市创建活动，提高市民文明素质。完善就业和社会保障体系。确保完成农村劳动力转移就业6万人，新增城镇就业15万人。深化和谐企业创建工作，健全完善企业职工工资集体协商、正常增长和支付保障机制。进一步做好社保扩面提标工作，实现新型农村社会养老保险制度全覆盖，完善被征地人员养老保障制度；加快发展老龄事业，抓好泉州老年公寓建设，多种形式兴建老年公寓，倡导居家养老，为广大老年人提供更多生活场所；实行工伤、生育、失业保险市级统筹，全面完成社保卡发放，提高新农合保障水平。进一步完善城乡低保和医疗救助制度，开展职工医疗互助活动，大力发展慈善事业，继续抓好社会救济、社会抚恤等工作。认真落实国家房地产调控政策，发挥住房公积金作用，建设各类保障性住房和改造危旧房1万套（户），建好东海保障性住房等项目，健全住房供应保障体系。做好市级救灾物资储备库和一批自然灾害应急避难场所建设。切实维护社会安定稳定。深化社会治安综合治理，深入推进社会矛盾化解、社会管理创新、公正廉洁执法三项重点工作，严密防范和严厉打击违法犯罪活动，扎实推进新一轮“平安建设”，做好信访工作，构建“大调解”工作体系。启动“六五”普法，加快依法治市进程。落实民族政策，依法管理宗教事务。强化食品药品监管，严厉查处制售假冒伪劣行为。实施“安全生产标准化建设三年行动”，深化专项整治，坚决杜绝重特大事故发生。深化双拥模范城创建活动。加强国防动员、民兵、预备役和海防、人防工作，认真落实拥军优属政策，妥善做好退伍、转业军人和随军家属优抚安置工作。

　　四、始终坚持雷厉风行，全面建设服务型政府

　　站在新起点、实现新跨越，是对政府建设一次全新考验。我们要以“等不起”的紧迫感、“慢不得”的危机感、“坐不住”的责任感，更加主动作为、高效作为、规范作为，更好地对人民负责、为人民服务、让人民满意。

　　要务实高效。始终保持雷厉风行、比学赶超的精神状态，推行“一线工作法”，到障碍最大的地方抓突破，到困难最多的地方抓协调，到任务最重的地方抓落实。推进县、乡政府机构改革，深化简政放权和简政强镇。加强网上审批系统建设，完善面向企业全程式网上审批、并联审批、绿色通道等制度。持续破解“熟人经济”，加大治庸治懒力度，深入推进政府绩效管理工作，营造一个适宜广大企业扎根发展的良好环境。要服务为民。视群众呼声为第一信号、群众需求为第一选择、群众满意为第一标准，把更多注意力放到社会管理上，把更多精力投入到公共服务上，把更多财力倾斜到改善民生上。广泛听取群众意见和建议，通过领导接访等形式，畅通社情民意反映渠道。抓好11件34项为民办实事项目，下大力气解决群众反映的热点难点问题，让广大群众共享发展成果。要依法行政。自觉接受人大法律监督、工作监督和政协民主监督，认真办理人大议案、代表建议和政协委员提案，充分听取各民主党派、工商联、无党派人士以及各人民团体的意见和建议，广泛接受新闻舆论和社会公众监督。深入贯彻《国务院关于加强法治政府建设的意见》，健全科学民主决策机制、常务会议学法制度，落实行政执法责任制，推进行政执法规范化建设，继续规范行政自由裁量权，深化行政指导，加强行政复议、仲裁工作，加大政府信息公开，设立行政权力电子监察中心。要廉洁从政。全体政府工作人员特别是领导干部一定要模范遵守《廉政准则》，牢记“52个不准”，推进惩防体系建设，认真落实党风廉政建设责任制，做到勤政为民、公道为政、廉洁为官，永葆政治本色，守住精神家园。加强述职述廉、审计监督和行政监察，突出重点领域、关键环节监督和制约，完善政府采购、产权交易、项目招投标等制度。坚持艰苦创业、勤俭办事，严格控制行政支出。建设一支政治坚定、业务精通、作风优良、廉洁高效的公务员队伍。

　　各位代表！回眸“十一五”，我们信心倍增；展望“十二五”，我们斗志昂扬。让我们在市委领导下，团结和依靠全市人民，弘扬泉州人爱拼敢赢、爱拼善赢、智拼会赢的豪迈气概，同心聚力，开拓创新，为推进泉州“两个加快”，实现科学发展、跨越发展而努力奋斗！
